# Interpretation of Heart Rate Variability: The Art of Looking Through a Keyhole

**DOI:** 10.3389/fnins.2020.609570

**Published:** 2020-12-21

**Authors:** John M. Karemaker

**Affiliations:** Department of Medical Biology, Section Systems Physiology, Amsterdam University Medical Centers, Amsterdam, Netherlands

**Keywords:** baroreflex sensitivity, biochip, cuffless blood pressure, non-linear analysis, preventive medicine, reticular activating system, science fiction, sympathovagal balance

## Abstract

The heart may be a mirror of the soul, but the human mind is more than its heart rate variability (HRV). Many techniques to quantify HRV promise to give a view of what is going on in the body or even the psyche of the subject under study. This “Hypothesis” paper gives, on the one hand, a critical view on the field of HRV-analysis and, on the other hand, points out a possible direction of future applications. In view of the inherent variability of HRV and the underlying processes, as lined out here, the best use may be found in serial analysis in a subject/patient, to find changes over time that may help in early discovery of developing pathology. Not every future possibility is bright and shining, though, as demonstrated in a fictional diary excerpt from a future subject, living in a society geared toward preventive medicine. Here implanted biochips watch over the health of the population and artificial intelligence (AI) analyses the massive data flow to support the diagnostic process.

## Introduction

### Diary Entries:

#### August 1, 2030, 10:30 a.m.

This morning I downloaded the data from my biochip to iCAIre, my home health system. It took longer than normal, which worried me, as always when the chip obviously had decided to do more measurements. A few days ago, iCAIre already made an appointment for me to see an eGP later today, we will have a videocall at 17:30 CET (Central European Time). The eGPs have their office in the United States, they came with the system that I chose. I could have checked in my preferences to have them contact my own GP directly, but hey, it is my health. I have looked at the files that were transmitted, but they did not make much sense to me: heart rate excursions, very large while I was asleep, same for blood pressure, oxygen- and CO_2_-levels. Probably I was tossing and turning very much while asleep. Would that explain my headaches, sleepiness and higher than normal blood pressure during the day? This biochip-thing is getting on my nerves. At the time it seemed like a good idea to have it implanted, my health insurance company insisted on it, or else my premium would almost double. So much for freedom of choice in the era of preventive medicine.

#### August 1, 2030, 20:30

That was not a nice videocall at all. The woman on the other side was rather blunt: I am overweight, my blood glucose is not OK, and, worst of all, I have developed obstructive sleep apnea, that is causing all this trouble during the night- and daytime. What now to do? See a dietician, I have to lose at least 20 kilos of my present 109. I always lived under the impression that my height of 1.85 m would allow me some more mass than average, but maybe she is right. In the meantime, I must make an appointment with my local GP whom they will contact to have me equipped with a device to prevent these apneic periods during sleep. I was reprimanded, because I should have checked in earlier, when the system started to bleep after every upload. In a way it was a lucky choice, this US system. It was more expensive than the regular ones, but it promised more privacy; the more common Asian systems are automatically coupled to the national registrar for preventive health care, my data are kept private as long as they ascertain that I am in the green zone. Which I, obviously, am not, at least not always. Now I risk to be exposed anyway. ICAIre will follow the developments closely, the operator said, not so much for my sake, but in order not to lose their license to sell and contract in the EU.

### Rationale

The title of the present Research Topic: *Horizon 2030: innovative applications of Heart Rate Variability* pushes the authors to, somehow, rise above their field and imagine a not-too-distant future where heart rate variability (HRV) might feature in a new role. The *Frontiers* journal is usually about science, but this invites now to venture the border between science and science fiction. Therefore, the present contribution is more a personal view rather than a systematic review of HRV interpretation. The paper’s title is to stress this fact: looking through a keyhole one can only observe part of what is going on behind that door and much of the testimony of what happened there is interpretation, maybe helped by knowledge of the players and what the room looks like, but not an actual eyewitness report.

## Heart Rate Variability

The use of HRV has expanded into many areas of biomedical research and even into medical apps on smartphones that are supposed to help a person relax. Still, in accepted medical practice HRV is at best a side note in a Holter monitoring report (i.e., a 24 h ambulatory ECG-recording). Why the discrepancy? Might this change in the coming decade? What do we understand about HRV, and how and when can that knowledge be of help in medical practice?

In theory, the origin of HRV is not elusive: generally speaking, fast, beat-to-beat variations in heart rate are parasympathetic, vagally mediated. The slower changes, extending over many beats, are mainly due to sympathetic nervous influences. Heart rate changes over long periods (tens of seconds to minutes and hours) are due to humoral factors like adrenaline, vasopressin and angiotensin (Karemaker, 2017).

## Specific Frequencies

Recognition of the underlying autonomic neural activities has awakened hope to understand the (central) nervous condition from heart rate variability. Initially, this was restricted mainly to measurement of respiratory variations (respiratory sinus arrhythmia, RSA), in search for a measure of vagal outflow. By maximal deep in- and expiration at a low frequency, it was held that vagal outflow was switched on and off, thus the peak-to-trough values of RSA would represent total vagal outflow. Decreases beyond those due to aging were (and are still) considered indicative of disease processes like diabetic neuropathy, as reviewed in Wieling et al. (1985). Later studies demonstrated the strict necessity of conditioning depth and frequency of respiration, as well as the level of exercise or sympathetic background activity, before making any inferences from RSA (Grossman et al., 1991).

Closer inspection of heart rate variations, aided by computer spectral analysis, had demonstrated the existence of slower than respiratory heart rate variations, in particular with around 10 s periodicity. These are also (mainly) due to vagal effects, but riding on slow sympathetically driven blood pressure waves (Sayers, 1971; Akselrod et al., 1981; DeBoer et al., 1987). Since the latter frequency, therefore, is related to sympathetic activity and the former, respiratory, to parasympathetic, the ratio between the powers in the two frequency bands LF/HF (LF, low frequency, i.e., 10 s rhythm, over HF, high frequency, respiratory rate) (Malliani et al., 1991; Camm et al., 1996) has developed into a number that is used to describe the balance in the central autonomic drive: sympatho-vagal balance. If we pause to think about this, attractive as it may be, it is obvious that this number comes with an intrinsic problem: both the fast and the slower heart rate variations are (mainly) vagally mediated and in most cases respiration depth and frequency are undefined. After administration of a vagolytic dose of atropine the time curve of heart rate is almost flat, only slow variations remaining. Likewise, during exercise the parasympathetic outflow is almost silenced, and no regular rhythms remain, neither around 0.1 Hz, nor respiratory. Still, no one would deny the existence of a strongly increased sympathetic state when heart rates go up to 150 beats per minute and higher.

## Variability Comes in Flavors

**FIGURE 1:**
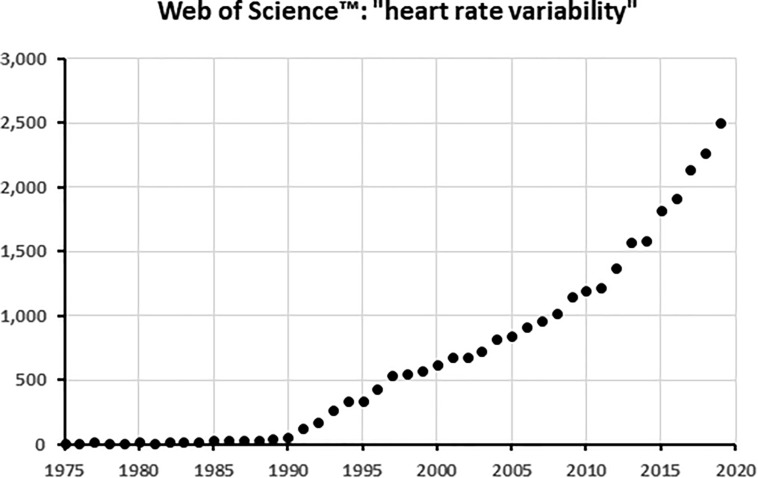
The number of articles with “Heart rate variability” in the title per year, as found in the Web of Science core collection^TM^ of scientific journals. When plotted on a logarithmic scale, the curve becomes an almost perfect straight line from 1996 onward, demonstrating its exponential increase.

In 1987 Kleiger and the multicenter post-infarction research group showed that decreased HRV in particular in the low- and very low-frequency area as calculated from 24 h ECG’s could predict mortality in the years after a myocardial infarction (Kleiger et al., 1987). A few years later, Bigger et al. (1993) demonstrated in a subset of the same patient population, that even 5–15 min recordings gave the same results with the same predictive power. These studies inspired from that time onward an almost exponential rise in publications related to HRV, as demonstrated in Figure 1 for the Core Journals in Web of Science^TM^. Of the around 30,000 core publications mentioning HRV up to now, only some 1,500 (5%) have “heart failure” as keyword as well. Much of the work is not so much devoted to practical medical applications as it is to the development of analysis techniques to squeeze information out of the HRV-signal. In particular, the easy availability of digitized long heart rate recordings has inspired the mathematics- and physics-savvy community to devise more and more intricate techniques of non-linear analysis to discover hidden features in HRV beyond those detectable by simple statistics of the signal in the time domain or by frequency domain analysis.

The first attempts were exploiting the fractal properties of the heart rate signal: the “jumpiness” that repeats itself on any scale, from seconds to minutes to hours (Goldberger, 1990). A true fractal signal shows up as a straight line when frequency and power are plotted on logarithmic scales (power law). And indeed, heart rate signals do (more or less). This inspired hope that the slope of that fractal line, the fractal dimension, would tell something about the condition of the test subject (di Renzo et al., 1996; Goldberger, 1996, 1997; Otsuka et al., 1997). However, that did not turn out so well: Eckberg and colleagues showed that the fractal dimension could change remarkably from day to day without any obvious reason in perfectly healthy subjects (Tan et al., 2009). On the other hand, Churchill et al. (2016) demonstrated that the fractal properties of the blood oxygen level dependent (BOLD) signal in fMRI are suppressed to variable degrees depending on the attention level required. If the resting level of brain activity is reflected in HRV, variable outcomes of resting HRV fractal exponent may be the result of (not-so resting) brain activity, unknown to the observer.

The next push came from application of information theory: a purely regular sequence of heart intervals has, information-wise, low entropy. From that insight various descriptors for the predictability, or rather unpredictability, have been proposed. The first application came by Pincus, who coined the term “Approximate Entropy” (ApEn) to describe the irregularity in a series of biological events like successive heart periods (Pincus, 1991; Pincus and Goldberger, 1994). Together with Goldberger he wrote a tutorial for physiologists in 1994 where they explained how this parameter should be calculated and interpreted. ApEn led to a number of follow-up and/or improved parameters: for one, Sample Entropy, introduced by Moorman to produce computationally more stable numbers, which also have more consistent outcomes when repeated in the same subject on different days (Richman and Moorman, 2000). Costa and Goldberger took the entropy analysis still one step further when they introduced multiscale entropy (MSE), where entropy calculations are done on “coarse grained,” time series i.e., resampled by packing more and more successive data points together to emphasize slower and slower trends in the signal (Costa et al., 2002, 2005).

The calculation of information entropy, applied to heart period series, has opened new doors to quantify the visual impression that one may have when looking at a beat-to-beat heart rate signal. It must be stressed, though, that there are thus far few practical applications. Moorman et al. devised a tool that has shown promise in neonatal intensive care units (Fairchild et al., 2013). Comparisons between more classical statistical- or frequency analysis derived- and entropy-derived measures do, however, not always favor the newer ones (Zhang et al., 2013). This may, partly, be due to the fact that in many applications, entropy-measurements require rather long recordings before reaching a stable value. In critical situations this is a serious drawback. However, when the requirement of more time and more data points it is not an issue, entropy analysis may be a viable option to dig deeper in the complexities of the heart rate signal at hand. The examples found in the literature demonstrate that it may point from changes in mood to underlying pathology, present or in the making (Ho et al., 2011). Therefore, the “dystopic diary entries” in the beginning of this paper point to a possible application: follow a person’s HRV over longer time to observe changes that might point to developing problems, like the diagnosis of obstructive sleep apnea during the night with its sequels in the daytime.

## Non-Invasive Continuous Blood Pressure

Heart rate should not be judged separately from the prevalent blood pressure (BP). The invention of a reliable method for non-invasive measurement of continuous BP by the Czech physiologist Jan Peňáz, further developed together with the Dutch biomedical engineer Karel Wesseling and his team (Penaz, 1973; Molhoek et al., 1984), has opened up a wide field of research by enabling riskless measurement in many test subjects under a large variety of circumstances. By the use of an inflatable finger cuff and concurrent measurement of the photoplethysmogram of the phalanx under study, a servo pressure loop keeps the pressure inside the cuff such that the vessels are continuously in a condition of almost-collapse. The only way to do that properly is by applying inside the cuff the same pressure as the one inside the vessels (give or take some pressure drop due to tissue pressure). This will reliably track intra-arterial pressure, if provisions inside the servo-machine are made to adjust for slow changes in the tissues of the compressed finger. This combination of ideas and engineering craftsmanship resulted in the successful introduction of the Finapres and the many varieties of the technique (Imholz et al., 1998). However, the cost of the necessary equipment has prevented its penetration into the consumer market; another drawback is its sensitivity to artefacts due to finger movement, to name but one.

Presently, cuffless blood pressure estimation is under way to fill that gap: by correlating upper arm BP, or some other calibrated pressure, to pulse wave velocity measurement. Pulse wave velocity in a vessel is related to its stiffness via the Moens–Korteweg equation: PWV = √(h.E/ρ.d), where h is wall thickness, d its diameter, E Young’s modulus (quantifying stiffness) and ρ the density of the fluid. Stiffness is related to wall composition, to the level of blood pressure and (strongly) to age. The method calculates BP by applying the estimated biophysical properties of the vascular wall in that particular subject to the arrival time of the pressure wave at e.g. the wrist after, for instance, the R-wave of the ECG (as such, it might even lend itself to incorporation of a biochip as mentioned in the introduction). A review of the method and various algorithms and calibration methods used is given in Sharma et al. (2017). The results have been quite variable until now, the method is not used in clinical monitoring devices due to its unreliability (Moharram et al., 2020). This has not prevented the marketing of devices for instance for sleep recording^1^ of blood pressure or 24 h continuous beat-to-beat estimation by the same principle, starting from one calibration with a traditional arm cuff. The issue has been picked up by the Institute of Electrical and Electronics Engineers (IEEE) that has developed its P1708-2014 – IEEE Standard for Wearable Cuffless Blood Pressure Measuring Devices. Even though that may sound like a precise way of testing some new device, the center piece remains the arm cuff, requiring two independent, trained observers who read the same sphygmomanometer using a Y-piece for their stethoscopes. In many hospitals and medical practices, doctors and nurses will prefer an automatic upper arm blood pressure measuring device. This works by the oscillometric principle and gives exact looking numbers for heart rate and systolic, diastolic (sometimes mean) to the nearest mmHg pressure value. The technique of accurate measurement by the hand-inflated arm cuff and stethoscope seems like a dying art.

In blood pressure measurement we seem to be stuck to a one century-old technique (Riva-Rocci cuff inflation, listening to the Korotkov sounds distal from the occluding point) expressing the pressure in a unit that no longer makes sense (mmHg) since mercury has long been banished from use due to its toxicity when spills are entering the environment. The various hypertension societies have successfully blocked the introduction of the International SI-unit of pressure, the Pascal (or rather the kilopascal in the case of BP), arguing that comparison to older literature and patient data will be hampered, which is a good point, but it postpones the shift forever and it makes for instance the understanding of flow resistance units so much more difficult.

## Baroreflex Sensitivity

The combined analysis of heart rate and continuous blood pressure adds another sought-after key figure to the non-invasive diagnostic and research arsenal, i.e., baroreflex sensitivity (BRS), the amount of heart period lengthening divided by the amount of causative blood pressure rise (ms/mmHg). BRS measurement, originally, was developed by imposing a fast blood pressure rise by the injection into the blood stream of a strongly vasoactive substance. At first this was mainly angiotensin (Smyth et al., 1969), later phenylephrine was predominantly used, when it became apparent that angiotensin has central effects on the vagal outflow to the heart. A thorough discussion of the use of bolus injections to measure BRS and the various drugs to do so is given in Eckberg and Sleight (1992).

Baroreflex sensitivity is the overall effect of a multi-step transduction process; at every step its effectiveness may be altered by factors external or internal to the reflex. It starts at the transformation of blood pressure changes to vessel wall extension, then to receptor activity to afferent nerve activity to nucleus tractus solitarii activity to efferent cardiac vagus nerve activity (and inhibition of sympathetic outflow) to sinoatrial node depolarization to atrial and then ventricular activation to arterial pressure pulse generation. Details of the various steps will be discussed below under the appropriate headings. This reflex loop is, of course, not only active when phenylephrine is injected, but continuously, from beat to beat. As a consequence, one may relate the changes in pressure to concomitant changes in heart period and expect to find a number related to the phenylephrine BRS. This, indeed proves to be the case: the correlation of the slower systolic pressure variations around 0.1 Hz to those in heart period gives numbers in the right order of magnitude (Robbe et al., 1987). The faster (HF-) variations give higher numbers, probably tainted by direct effects of the respiratory centers on vagal outflow to the heart (Frederiks et al., 2000). The numbers found are not perfect, they tend to come with a sizeable variability, but so does BRS by the phenylephrine method; the output via the vagus nerve is rather variable.

The variability of BRS comes to light by a technique that follows the baroreflex, as it were, from moment to moment while comparing sliding windows of 10 s blood pressure values to ensuing heart periods (xBRS). This gives very variable numbers over short periods, but the geometric averages correlate favorably to the BRS determined by vasoactive drug injections in the same experimental subjects (Wesseling et al., 2017). The same variability is found when short segments are searched in BP-HR recordings, to measure a valid baroreflex slope when sufficient correlation is detected [the “slopes-technique,” cf. (di Rienzo et al., 1996)]. The inherent variability of beat-to-beat blood pressure control has led (Wessel et al., 2020) to take the position that these methods only look at variability ratios between blood pressure and heart rate. Probably that is exactly what the baroreflex should do: exchange blood pressure variability for heart rate variability. The former is the value to be kept between boundaries for proper functioning of the body, the latter is, within limits, less of a consequence (Karemaker and Wesseling, 2008).

## Afferents That May Alter Heart Rate

Considering all incoming nervous activity to the CNS, ultimately any afferent nerve may have an effect on HR. Not all nerves will have an equally strong or immediate effect, but, if the stimulus is sufficiently strong, this corollary will hold true. Importantly, visceral afferents from thoracic or abdominal organs can elicit large HR-effects, mainly by their ability to modulate outgoing vagal activity to the heart. This occurs at a fairly basal level, just above the spinal cord, in the medulla oblongata (Guyenet, 2006). There, the nucleus of the solitary tract (NTS, nucleus tractus solitarii) is receiving these inputs. Not all afferents, be they sensory (e.g., touch, pressure, and pain), proprioceptive (e.g., muscle and tendon) or visceral (e.g., stretch of the intestinal wall) evoke heart rate changes to the same extent, or even in the same direction, up or down. One way how the incoming afferent information may evoke these heart rate responses is by blocking the transfer from baroreceptor inputs to cardiac vagal efferent output. This has been shown in very elegant experiments by Iriuchijima (Iriuchijima and Kumada, 1964) who stimulated baroreceptor afferent nerves and concurrently afferents from muscles or skin, while looking at the reflexly induced cardiac vagal activity. In human subjects it also appeared to be the best explanation for the peripheral part of the “muscle-heart” reflex, where a single bout of muscular exercise can induce an immediate heart rate increase. The same effect was provoked by electrical stimulation of the nerve to have the (arm-) muscle contract, but not when the vagus nerves had been blocked by atropine (Hollander and Bouman, 1975).

Afferent information from the region of the head, incoming *via* the cranial nerves, is not special in this respect: this, too, may give rise to heart rate effects when activated, for this a descending pathway down the brain stem to the medullary level exists. Some specific afferent activity may even trigger early evolutionary mechanisms, in particular the diving response can be mentioned here in response to cold wetting of the face (Folkow et al., 1971). On the other hand, input from the “special senses” (taste, smell, sound, and sight) is so much intertwined with emotional cues from earlier ingrained memory trails that one cannot predict the autonomic effects. The same holds true for the C-fiber afferents that are specifically sensitive to stroking or caressing between subjects. For instance, depending on who seems to be doing the caressing to an infant, the heart rate will decrease more if this is done by the daily caregiver [although, unseen by the infant, the caressing was always done, in the experiment, by the same “stranger” (Aguirre et al., 2019)].

## Central Mechanisms: Wake-Sleep and Alertness

Alertness and wake-sleep state are maintained by a dispersed system of nuclei extending from the lower medulla oblongata all the way to the upper part of the midbrain. These nuclei are interconnected like a network, hence its name: the reticular formation, i.e., the Network, also known as the ascending reticular activating system or ARAS. Its activity is a requirement for consciousness and to keep the higher centers of the brain awake [for a critical review of the concept see Berlucchi and Marzi (2019)]. The relation between ARAS and HRV is unexplored territory; for functional imaging the number of active neurons is relatively low and the medulla/brainstem system is difficult to lift out of the noise.

In view of the difficulties of investigating the brainstem and related structures, only few researchers in brain fMRI have made the central autonomic system their field of interest. Still, in the last one and a half decade or so good progress has been made, while tracing non-invasively in awake, healthy humans the path of sympathetic and parasympathetic control of the circulation. In the first place a group around Macefield and Henderson in Australia is to be mentioned who, in a clever experimental design, tracked the origin of simultaneously recorded peripheral sympathetic nerve activity to the level of the hypothalamus and to higher loci in the cortex (Macefield et al., 2013). A Canadian group around K. J. Shoemaker focused more on the cortical and directly subcortical centers involved in blood pressure and heart rate control (Cechetto and Shoemaker, 2009; Ruiz Vargas et al., 2016). Finally, a group in Germany, combining researchers from various institutes must be mentioned (Gerlach et al., 2019; Manuel et al., 2020). In their last study they provoked hypotensive responses by enclosing the lower body of the test subjects in a box and applying suction to induce a situation that may be compared to hemorrhage (or prolonged standing). In an earlier study they looked at the path of baroreceptor information at blood pressure increases by injection of phenylephrine. From the combined analyses of these groups we may start to construct a model of the brain stem nuclei involved in control of the cardiovascular system, managing vagal, sympathetic and humoral outputs like the antidiuretic hormone. This, effectively confirms in awake humans the wealth of earlier animal studies relating brain stem nuclei to cardiovascular control. Moreover, some “higher” centers also join into this framework, making the link to what has become known as the Default Mode Network, i.e., the ongoing cortical activity, ranging around when the mind is wandering in a resting, but awake condition.

## Final Common Automatic Pathway: The Vagus Nerve and Sympathetics

The healthy nervous system is never silent. Even while meditating, trying to stop the thoughts from running around and “emptying the mind,” techniques like EEG and fMRI will show that whatever the self may perceive as emptiness, nonetheless the central nervous system is active and not just in basic life support for respiration and blood pressure. Thereby, radiating out from this activity of the default mode network, vagal and sympathetic nervous outflow may still be modulated, leading to heart rate variability. The basic level of activity of either system is set by the demands of the moment (rest or activity), dictated by higher centers enforcing a perception of the present condition: flight or fight, stress or relaxation or something in between. In this personal review I will not go into the origins and specifics of the “polyvagal theory” (Porges, 2011) but see Grossman and Taylor (2007). One may take a practical view about this: if it works for psychotherapy to help patients understand and manage their innermost feelings, then the scientific merits of the theory are irrelevant.

## The Heart as Recipient of Incoming of Incoming Autonomic Information

Final output from the CNS to the heart comes from the nucleus ambiguous in the medulla oblongata as far as the vagal activity is concerned and from the sympathetic command neurons in the rostroventrolateral medulla. The axons of the latter descend to the sympathetic motor neurons in the intermediolateral columns at the lowest cervical and upper thoracic segments of the spinal cord. However, the simple pictures from the textbooks as to how the final, peripheral postganglionic fibers reach their target require an update. Close to the heart the autonomic fibers form intricate plexuses, together nicknamed the heart’s “little brain” (Armour, 2008), like the elaborate autonomic nervous system that regulates all of intestinal function from within as reviewed in Karemaker (2017).

Nervous activity in this interwoven network of sympathetic and parasympathetic fibers will, finally, lead to heart rate variability, or rather to liberation of autonomic transmitters at the sinus node. Its effect is not exactly predetermined by the type (adrenergic or cholinergic) and amount, but dependent on the moment of arrival in the cycle of the sinus node and on the previous history of autonomic stimuli. In an earlier study (Karemaker, 2015). I showed that the time of arrival of a burst of vagal activity, although beat-by-beat exactly identical, can be such, that the ensuing heart rate is fairly irregular. On the other hand, if the same bursts were applied earlier or later, heart rate would be very stable. If the vagal bursts were diminished by one stimulus only, near the unstable region one would see much more effect on the ongoing heart period than in the stable regions.

## Discussion: What Does Heart Rate Variability Tell?

Apart from heart rate variations due to some form of cardiac disease, which have been left out of the equation altogether in this review, one should maybe better ask “what does HRV not tell?” In the literature a wide range of conditions, healthy and diseased, has been explored, where HRV in some way would be different, at the group level, from a reference group. As I tried to demonstrate in the above, so many different circuits are involved in the stream of information reaching the motor neurons in the medulla and finally the heart, that it is next to impossible to retrace at any given moment what may have caused this particular output. Even quasi simple effects, like respiratory sinus arrhythmia, may turn out very differently in the same subject under slightly different circumstances.

Therefore, as hinted in the beginning, tracking a person’s HRV over time may better tell a story where pathology is developing. We have demonstrated something along those lines when we looked at HRV in pregnant women who developed pre-eclampsia (Rang et al., 2004). In yet another study we have shown that those who go on to develop full blown rheumatoid arthritis have higher resting heart rates and lower parasympathetic activity expression levels on circulating monocytes than a control group (Koopman et al., 2016). This conclusion may seem at variance with fMRI imaging studies where definite relations between centrally induced states of stress or relaxation were found at loci that subsequently were labeled sympathetic or parasympathetic with changes in HRV-patterns (Guyenet, 2006; Thayer et al., 2012; Beissner et al., 2013; Churchill et al., 2016). However, that does not make the reverse true: the relation between some (changed) HRV and a specific central condition is ambiguous. The various physiological processes involved between a primary emotional condition and what, finally, results in HRV, or autonomic nervous outflow for that matter, are too numerous. They may all leave their fingerprints, making the effect at the target organ uninterpretable. There are good reasons to mistrust the outcome of a lie detector test, as a combination of autonomic nervous responses to an emotional challenge. Therefore, this paper is intended to stress the importance of serial measurement in a subject if conclusions are to be based on autonomic measures like HRV. The measurements from an implanted biochip as mentioned in the beginning will make sense when they are followed over longer time periods, while measuring more than just one parameter. The day-to-day variability will be canceled out and deep learning algorithms may extract recurring features important for healthcare. The sheer amount of data that will be produced this way, obviously necessitates automatic data collection and artificial intelligence techniques to make sense of this. The present use of smartwatches is leading the way in this direction by applications like out of hospital detection of atrial fibrillation attacks or the cardiovascular status of chronic heart failure patients (Wang and Zhou, 2019; Wasserlauf et al., 2019). However, the diary entrances in the beginning of this paper may serve as warning signs, underlining the need for a discussion on “digital healthcare” that is gradually and silently taking control of our lives (Lupton, 2015).

## Author Contributions

The author is the sole creator of this manuscript. All views expressed here are entirely his own responsibility.

## Conflict of Interest

The author declares that the research was conducted in the absence of any commercial or financial relationships that could be construed as a potential conflict of interest.

## Footnotes

1 https://somnomedics.de/en/solutions/blood-pressure/somnotouch-nibp/
